# Assessment of lung function variability documents airflow limitation in many patients with long covid

**DOI:** 10.1016/j.heliyon.2024.e29261

**Published:** 2024-04-06

**Authors:** Tonje Reier-Nilsen, Charlotte Grønneberg, Stephanie Røine, Björn Nordlund

**Affiliations:** a The Norwegian Sports Medicine Centre – Football Association, Oslo, Norway; b The Norwegian Olympic Sports Centre, Norwegian Olympic and Paralympic Committee and Confederation of Sports, Oslo, Norway; c Department of Women's and Children's Health, Karolinska Institutet, Stockholm, Sweden; d Astrid Lindgren Children's Hospital, Stockholm, Sweden

**Keywords:** App-based spirometry, Case report, Postviral asthma, Lung function variability, Long covid

## Abstract

**Background:**

It is estimated that 65 million people worldwide suffer from long covid (LC). Many LC symptoms are also reported by patients with airflow limitation, used to confirm asthma. The primary aim was to detect airflow limitation in LC patients by a methacholine bronchial provocation test (BPT) and if negative, by evaluation of diurnal variability in forced expiratory flow in 1 second (FEV_1_) over a two-weeks’ period. The second aim was to assess responsiveness to asthma treatment on diurnal FEV_1_ variability and LC symptoms.

**Methods:**

Patients with LC for at least six months were recruited in this open diagnostic study. Burden of LC symptoms were reported on a 10-point Likert scale (0 = not troubled, 10 = extremely troubled) at inclusion and after three weeks’ asthma treatment. A positive methacholine BPT was defined by an accumulated provocation dose (PD_20_)<8 μmol causing 20% fall in FEV_1_. App-based spirometer was used for diurnal FEV_1_ variability, deemed positive by diurnalvariability in FEV_1_ ≥12%.

**Results:**

Airflow limitation was documented by positive methacholine BPT in 8/30 (27%), or by excessive diurnal variability in FEV_1_ in 21/22 (95%) of the BPT negative LC patients. One patient dropped out due to personal issues. Three weeks’ asthma treatment normalised mean diurnal FEV_1_ variability from 18.0% to 7.3%, p < 0.001. Significant reductions were observed for fatigue and dyspnoea, from 8.3 to 6.1, p < 0.001, and 3.0 to 0, p < 0.001, respectively.

**Conclusion:**

This study indicate that airflow limitation may be detected in many LC patients if evaluation of diurnal variability in FEV_1_ is included in the diagnostics.

## Abbreviations

BPT –: bronchial provocation test
CI -: confidence interval
COVID-19 -: coronavirus disease 2019
FEV_1_ -: forced expiratory volume in 1 s
GINA -: The Global Initiative for Asthma
LC –: long covid
PD_20_ -: provocation dose causing 20% fall in forced expiratory flow ventilation in 1 second
SARS-CoV-2 -: severe acute respiratory syndrome coronavirus 2

## 1 Introduction

The World Health Organization has urged action since an estimated 65 million people worldwide suffer from long covid (LC) [1,2]. Long covid is defined as a condition of persisting symptoms for at least 3 months after infection with the severe acute respiratory syndrome coronavirus 2 (SARS-CoV-2) [2]. Even though an infection with SARS-CoV-2 results in mild coronavirus disease 2019 (COVID-19) in approximately 80% of the cases [2], up to 30% report persistent symptoms regardless of COVID-19 severity [3]. The most common symptoms reported by 40–70% of patients with LC are fatigue, shortness of breath/dyspnoea and muscle pain/post-exertional malaise, as well as brain fog, headache, memory issues, tachycardia and [[3], [4], [5], [6]]. Numerous different mechanisms have been studied to explain LC, varying from psychological to biological mechanisms including clotting/coagulation issues and disrupted brainstem/vagus nerve signaling, among others [7]. However, during a study including field-based sport-specific exercise tests in athletes where app-based spirometry was performed at home before and after each exercise [8], we observed several cases where lung function given as forced expiratory volume in 1 s (FEV_1_) decreased significantly after vaccination with SARS-CoV-2 or after COVID-19. This airflow limitation is a common complication of respiratory tract infections, induced by postviral inflammation with subsequent airway narrowing [9], commonly referred to as postviral bronchial hyperreactivity [9]. This condition is self-limited resolving within a 5–11 weeks duration time [10], in contrast to asthma, which is a chronic condition (>3 months) also associated with airflow limitation as a consequence of inflammation. Typical respiratory symptoms include wheezing, cough, chest tightness/dyspnoea and excessive mucus, which vary by time depending on the varying inflammation [11]. Airflow limitation may also induce compensatory hyperpnoea with tachycardia [12], and hypoventilation with subsequent non-respiratory symptoms like fatigue, headache and memory issues [13], as well as post-exertional malaise and muscle aches [14]. The diagnosis of asthma is confirmed by detection of the airflow limitation, in practice by laboratory bronchial provocation tests (BPTs), but evaluation of diurnal variability in lung function may also be used in this manner, according to GINA - The Global Initiative for Asthma [11]. Except for individual case reports [15], we are unaware of previous studies that have been able to detect airflow limitation as a possible explanation of LC symptoms [6,7]. In this open diagnostic pilot study, the primary aim was to detect airflow limitation in LC patients, first by a methacholine BPT, and if negative, by evaluating diurnal variability in lung function by using a remote app-based spirometer over a two-weeks’ period [11]. The second aim was to assess responsiveness to asthma treatment on diurnal variability in lung function and LC symptoms in patients with confirmed airflow limitation.

## 2 Methods

### 2.1 Study design and study population

Patients with LC for at least six months were invited to participate in this open diagnostic study. They were referred from primary care for physical rehabilitation at the Norwegian Sports Medicine Centre – Football Association in Oslo, Norway. The rehabilitation program included easy mat activities two to three times per week. Participants were consecutively recruited and not selectively chosen beyond the initial criteria, and there were no exclusion criteria, except for the requirement to comply with lung function test.

The study was approved by the Regional Committee for Medical and Health Research Ethics in Oslo, Norway (number 535147) and registered by ClinicalTrials.gov number NTC05919004.

### 2.2 Data collection and outcomes

A survey was developed to capture self-reported burden of LC symptoms including 10-point Likert scales (0 = not troubled, 10 = extremely troubled) for shortness of breath/heavy breathing, fatigue, headache, brain fog, memory loss, concentration issues, loss of taste, loss of smell, tachycardia, chest pain, muscle pain, post-exercise malaise, dizziness, anxiety, depression, sleep disturbances, increased sensitivity to sound and light, indigestion. The survey was completed at inclusion and after three weeks of eventual asthma treatment.

A methacholine BPT was performed using an inspiration-triggered nebulizer (MasterScreen Pneumo Jäger, Würzburg, Germany) to determine the accumulated provocation dose (PD_20_) causing a 20% fall in forced expiratory volume in 1 second (FEV_1_), according to guidelines [16]. The methacholine BPT was deemed positive by a PD_20_ < 8 μmol [16].

The methacholine BPT-negative patients were instructed to download the CE-marked AsthmaTuner app on their iOS or Android smartphone (MediTuner, Stockholm, Sweden). The cloud-based system of AsthmaTuner [17] includes 1) a handheld validated Bluetooth turbine-spirometer (MIR, Rome, Italy) and 2) app-based software with a web-interface. Spirometry results including variability (%) are presented in real-time to the clinician's web-interface [17]. The built-in software performs quality controls of the spirometry manoeuvres in line with guidelines [16], enabling that FEV_1_ is based on a maximal effort and requiring a minimum of three acceptable manoeuvres for an approved spirometry test [16]. The patients were educated by a clinician to perform lung function tests on the app-based spirometer at study inclusion until the proper technique was achieved. Remote app-based spirometry was performed by the patients every morning and evening, and before and within 10 minutes post mat activities in the rehabilitation program. Excessive lung function variability was defined as ≥12% diurnal variability in FEV_1._ [11].

Subjects with either a positive methacholine BPT or excessive diurnal variability in FEV_1_ were defined with airflow limitation and received asthma treatment with inhalation corticosteroids and eventual long-acting beta_2_-agonists and/or long-acting muscarinic antagonists in line with guidelines [11]. After three weeks, the patients were re-evaluated for LC symptoms and variability in lung function.

Statistical analyses were performed with Ledidi Core (Ledidi, Oslo, Norway), using non-parametric Mann–Whitney test.

## 3 Results

At inclusion, all thirty patients (median age 41 yrs, range 24–56, 73% females) were on partial or full occupational sick leave and gave written informed consent for participation. Self-reported clinical allergy was reported by 60% and previous asthma diagnosis by 47%, but only 17% used asthma medication including inhalation corticosteroids when they first had their COVID-19 nor later in their LC course, due to self-perceived non-symptomatic asthma (Table 1).

**Table 1:** Baseline characteristics of patients with long covid for at least six months referred for physical rehabilitation with easy mat activities.

Age	Median (range)	41 (24–56)
		n (%)
**Female gender**		22/30 (73.3)
**Allergy**		18/30 (60.0)
**Previous asthma diagnosis**		14/30 (46.7)
**Asthma medication when COVID-19**		5/30 (16.7)

* Self-reported clinical allergy.

**Use of asthma medications including inhalation corticosteroids.

A history of mild COVID-19 was reported by 29/30, while only one had been hospitalized. All patients completed the 10-point Likert scale on perceived burden of LC symptoms The highest scores were observed for fatigue, mean 8.3 (95% confidence interval (CI): 2.0, 10.0), memory loss 6.3 (95% CI: 0.0,10.0) and headache 5.2 (95% CI: 0.0, 10.0), while 16 patients reported shortness of breath/heavy breathing with mean 3.0 (95% CI: 0.0, 10.0). (Supplementary Figure).

**Fig. 1:**
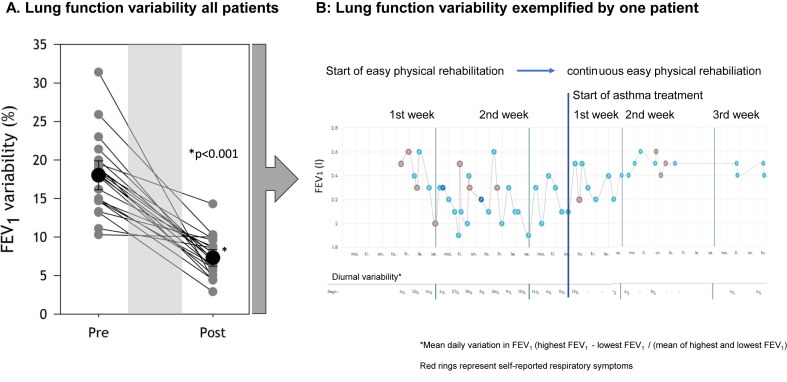
**1A.** Time course of mean FEV_1_ variability ([FEV_1_ highest – FEV_1_ lowest]/[mean of highest and lowest FEV_1_]) (%) from the two weeks' assessment period to the third week of asthma treatment, with a p-value of <0.001. Grey symbols represent each patient, while black symbols represent mean FEV_1_ variability (%) with a 95% confidence interval for all patients, **1B**. Typical time course of FEV_1_ exemplified by one patient. FEV_1_ - forced expiratory flow ventilation in 1 second.

Airflow limitation was documented in 29 of the 30 patients, in which the methacholine BPT was positive in 8 of 30 (27%) patients, while 21 of the 22 methacholine BPT negative patients demonstrated excessive variability in FEV_1_ (Fig. 1). An average diurnal variability in peak expiratory flow (PEF) > 10% was not observed [11]. Excessive variability in FEV_1_ became successively more apparent during more days of physical rehabilitation (Fig. 1). One patient dropped out of the study due to personal issues. Among the 29 LC patients with airflow limitation, 16 had no previous asthma diagnosis and 5/16 (31.3%) of these had clinical allergy as a predisposition for airflow limitation consistent with a new asthma diagnosis.

The mean FEV_1_ variability ([FEV_1_ highest – FEV_1_ lowest]/[mean of highest and lowest FEV_1_]) changed significantly from 18.0% (96% CI: 10.3, 31.4%) during the two weeks’ assessment period prior to 7.3% (95% CI: 2.9, 14.3%), p < 0.001 during the third week of asthma treatment (Fig. 1). After three weeks of asthma treatment, significant changes on the 10-point Likert scale on self-reported burden of LC symptoms were observed for fatigue from 8.3 (2.0, 10.0) to 6.1 (1.0, 10.0), p < 0.001, and for shortness of breath from 3.0 (0.0, 10.0) to 0 (0.0, 0.0), p < 0.001.

## 4 Discussion

In this study of patients with LC for at least six months, 29/30 patients either had a positive methacholine BPT (n = 8/30) or excessive diurnal lung function variability (n = 21/22) during two weeks of easy physical activity, consistent with airflow limitation. Consequently, these 29/30 LC patients received asthma treatment, whereafter three weeks’ treatment resulted in normalisation of diurnal lung function variability, absence of dyspnoea and significant reduction of fatigue.

These findings support that airflow limitation associated with asthma may play a role in patients with LC regardless of a predisposition like previous asthma diagnosis or clinical allergy. Even though 14/30 of the LC patients had a previous asthma diagnosis, only five patients used asthma medications when they got COVID-19, due to no self-perceived symptoms of asthma**.** Hence, most of the LC patients (n = 25/30) regarded themselves as healthy or not having a chronic respiratory disease requiring treatment at the time of their SARS-Cov-2 infection. Additionally, only 5/16 (31.3%) of the patients had clinical allergy as a predisposition for airflow limitation consistent with a new asthma diagnosis.

The diagnosis of airflow limitation may be complicated by the heterogeneity of LC symptoms, where non-respiratory symptoms often are referred to as the most burdening LC symptoms [6]. This is in line with the findings in this study, where shortness of breath/heavy breathing was only reported by 16 patients and at a low Likert score of 3.0. However, we observed successively increasing variability in lung function during the two weeks of only easy mat activities. This finding may explain why easy physical activity is reported to increase dyspnoea as well as other LC symptoms in LC patients [18]. It might be that non-respiratory symptoms may become more dominant than dyspnoea since LC patients often have reduced their physical activity to a minimum.

The airflow limitation in 29/30 of our LC patients was documented by a positive methacholine BPT in 8 of 30 (27%) patients, and excessive variability in lung function by FEV_1_ in 21 of the 22 methacholine BPT negative patients. Hence, the diurnal variability of FEV_1_ gave valuable additional information on a present airflow limitation in approximately 2/3 of the LC patients. This is in line with reports from a recently published study [8], where as much as 59% of 41 athletes with asthma had a negative methacholine BPT and/or eucapnic voluntary hyperpnoea, and airflow limitation was only detected on home spirometry before and after exercise. The documented airflow limitation in our 29/30 LC patients, may explain why systemic [19,20], as well as inhaled corticosteroids [21,22], are observed to be effective in LC patients. Recently, findings of continuous elevated inflammation markers in LC patients may explain why the airflow limitation does not seem to be self-limiting within the expected 5–11 weeks [10], supporting the potential of a persisting condition.

In the present study, three weeks of asthma treatment resulted in objective normalisation of diurnal lung function variability by regularly home spirometries, as well as self-reported absence of dyspnoea and significant reduction of fatigue. In our population, fatigue, memory loss and headache were the most dominant LC symptoms, and the patients reported significant reduction in fatigue after only three weeks of asthma treatment. This may be explained by the association between asthma and non-respiratory symptoms, such as fatigue, headache and memory issues [13]. Other non-respiratory symptoms associated with asthma are also reported by LC patients, including tachycardia [12], as well as post-exertional malaise and muscle aches [14]. Hence, we suggest that airflow limitation should be assessed by daily monitoring of lung function regardless of reported respiratory symptoms in LC patients. Furthermore, daily monitoring of lung function during physical rehabilitation in LC patients is recommended to diagnose otherwise undetected airflow limitation.

The current study faces potential limitations of a small and selected cohort and the use of unsupervised app-based lung function tests. However, the reliability of the method is secured by the strict built-in ATS/ERS guidelines which are reported to reject more manoeuvres than human reviewers [16,23]. The study does not include a control group, but the results are strengthened by the objective normalisation in lung function variability detected by the app-based spirometer.

## 5 Conclusion

Daily monitoring of lung function in LC patients may aid in detecting excessive lung function variation consistent with airflow limitation associated with asthma. In the face of limited diagnostic and treatment recommendations, diagnostic evaluation of LC patients could benefit from a broad evaluation of airflow limitation including diurnal lung function with FEV_1_. We suggest that future interventional studies should focus on asthma treatment effects in LC patients with evidence of airflow limitation.

## Data availability statement

Data included in this study have not been deposited into a publicly available repository. In the case of science projects, data may be available on request.

## Ethics declarations

The study was approved by the Regional Committee for Medical and Health Research Ethics in Oslo, Norway (number 535147) and registered by ClinicalTrials.gov number NTC05919004. All participants gave written informed consent to the participation of this study and for the publication of their anonymised case details.

## Guarantor statement

TR-N confirms full responsibility for the content of this manuscript.

## Declaration of generative AI in scientific writing

Artificial intelligence has not been used in the writing process.

## Funding source

None.

## Ethics committee approval

The study was approved by the Regional Committee for Medical and Health Research Ethics in Oslo, Norway (number 535147).

## Clinical trial registration

ClinicalTrials.gov number NTC05919004.

## Clinical implication

Daily monitoring of lung function in patients with long covid may aid to detect excessive lung function variation consistent with asthma. An app-based spirometer may potentially be used for illness surveillance and to assess response to treatment.

## CRediT authorship contribution statement

**Tonje Reier-Nilsen:** Writing – review & editing, Writing – original draft, Validation, Software, Project administration, Methodology, Investigation, Formal analysis, Data curation, Conceptualization. **Charlotte Grønneberg:** Writing – review & editing, Investigation, Data curation. **Stephanie Røine:** Writing – review & editing, Data curation. **Björn Nordlund:** Writing – review & editing, Validation, Methodology.

## Declaration of competing interest

Tonje Reier-Nilsen is employed as part-time consultant in MediTuner. Charlotte Grønneberg and Stephanie Røine have no conflicts of interest. Björn Nordlund was part of founding MediTuner AB, the company owning the medical device AsthmaTuner.
