# Supplementary figures and images for: Assessment of lung function variability documents airflow limitation in many patients with long covid
Source: Heliyon. 2024 Apr 6;10(8):e29261. doi: 10.1016/j.heliyon.2024.e29261 (PMC11019201; doi:10.1016/j.heliyon.2024.e29261)

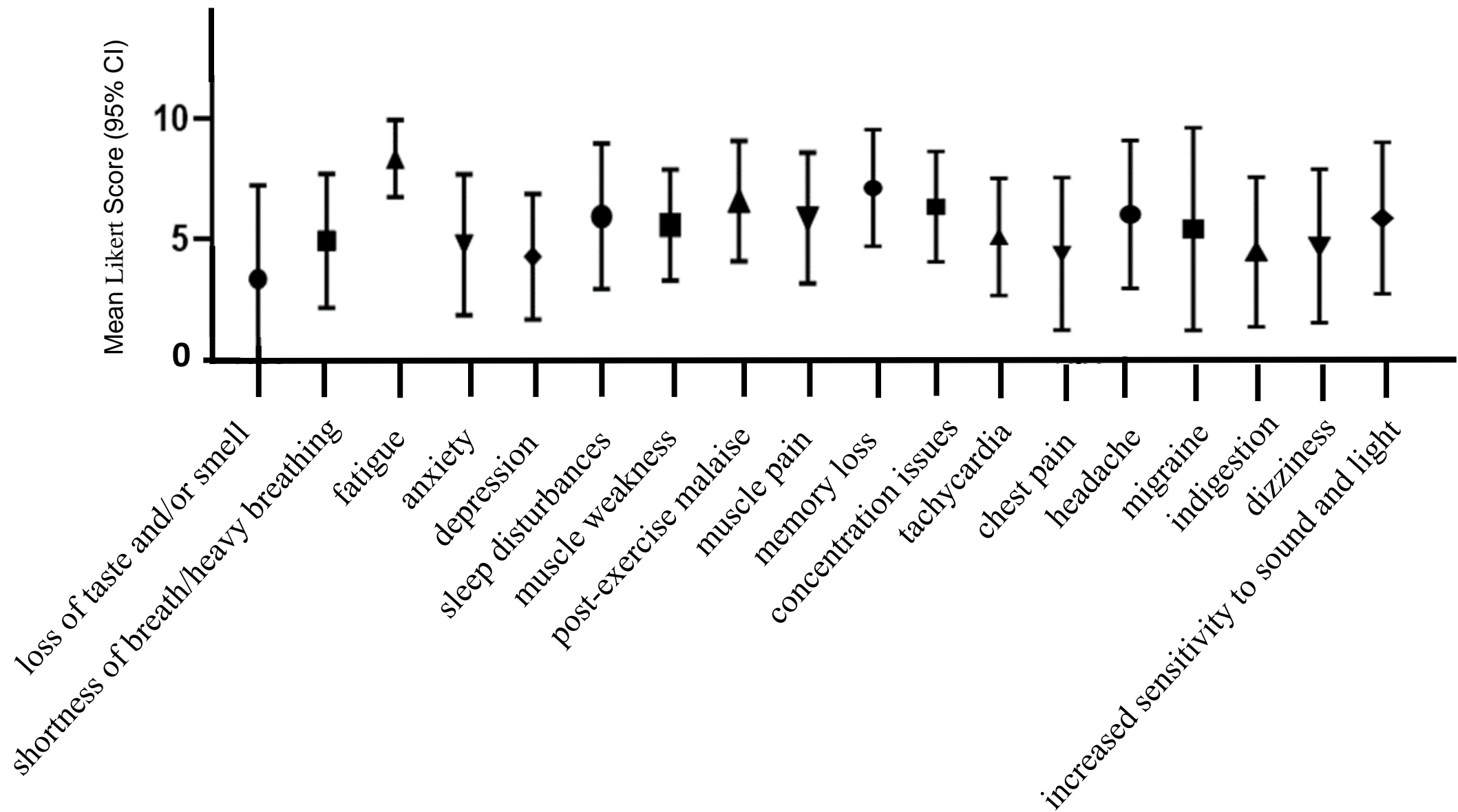

Supplement: Multimedia component 1 [file mmc1.pdf]
